# Modeling undetected live poliovirus circulation after apparent interruption of transmission: implications for surveillance and vaccination

**DOI:** 10.1186/s12879-015-0791-5

**Published:** 2015-02-18

**Authors:** Dominika A Kalkowska, Radboud J Duintjer Tebbens, Mark A Pallansch, Stephen L Cochi, Steven G F Wassilak, Kimberly M Thompson

**Affiliations:** Kid Risk, Inc., 10524 Moss Park Road, Site 204-364, Orlando, FL 32832 USA; Delft University of Technology, Delft, Netherlands; Division of Viral Diseases, National Center for Immunization and Respiratory Diseases, Centers for Disease Control and Prevention, Atlanta, GA USA; Global Immunization Division, Center for Global Health, Centers for Disease Control and Prevention, Atlanta, GA USA; College of Medicine, University of Central Florida, Orlando, FL USA

**Keywords:** Disease outbreaks, Disease transmission, Models, Statistical, Poliomyelitis, Poliovirus, Surveillance, Risk assessment, Vaccination

## Abstract

**Background:**

Most poliovirus infections occur with no symptoms and this leads to the possibility of silent circulation, which complicates the confirmation of global goals to permanently end poliovirus transmission. Previous simple models based on hypothetical populations assumed perfect detection of symptomatic cases and suggested the need to observe no paralytic cases from wild polioviruses (WPVs) for approximately 3-4 years to achieve 95% confidence about eradication, but the complexities in real populations and the imperfect nature of surveillance require consideration.

**Methods:**

We revisit the probability of undetected poliovirus circulation using a more comprehensive model that reflects the conditions in a number of places with different characteristics related to WPV transmission, and we model the actual environmental WPV detection that occurred in Israel in 2013. We consider the analogous potential for undetected transmission of circulating vaccine-derived polioviruses. The model explicitly accounts for the impact of different vaccination activities before and after the last detected case of paralytic polio, different levels of surveillance, variability in transmissibility and neurovirulence among serotypes, and the possibility of asymptomatic participation in transmission by previously-vaccinated or infected individuals.

**Results:**

We find that prolonged circulation in the absence of cases and thus undetectable by case-based surveillance may occur if vaccination keeps population immunity close to but not over the threshold required for the interruption of transmission, as may occur in northwestern Nigeria for serotype 2 circulating vaccine-derived poliovirus in the event of insufficient tOPV use. Participation of IPV-vaccinated individuals in asymptomatic fecal-oral transmission may also contribute to extended transmission undetectable by case-based surveillance, as occurred in Israel. We also find that gaps or quality issues in surveillance could significantly reduce confidence about actual disruption. Maintaining high population immunity and high-quality surveillance for several years after the last detected polio cases will remain critical elements of the polio end game.

**Conclusions:**

Countries will need to maintain vigilance in their surveillance for polioviruses and recognize that their risks of undetected circulation may differ as a function of their efforts to manage population immunity and to identify cases or circulating live polioviruses.

**Electronic supplementary material:**

The online version of this article (doi:10.1186/s12879-015-0791-5) contains supplementary material, which is available to authorized users.

## Background

The Global Polio Eradication Initiative (GPEI) continues to make progress toward eliminating all paralytic cases caused by wild polioviruses (WPVs). The GPEI primarily relies on routine immunization (RI) and supplemental immunization activities (SIAs) using the live, attenuated oral poliovirus vaccine (OPV), [1,2] a comprehensive surveillance system, and aggressive outbreak response [3,4]. Typical estimates suggest that permanent paralytic poliomyelitis may occur at a ratio of approximately 1 case per 200 WPV serotype 1 (WPV1) infections on average in immunologically naïve (i.e., fully susceptible) individuals [2,5], with lower ratios around 1 in 2000 and 1 in 1000 for WPV2 and WPV3, respectively [5-7]. Previously vaccinated or infected individuals can also become asymptomatically infected and potentially participate in poliovirus transmission despite remaining protected from paralytic poliomyelitis, [7-9] which effectively decreases the overall apparent paralysis-to-infection ratio for the entire population. The use of OPV leads to a very small fraction of vaccine recipients (or their close contacts) developing vaccine-associated paralytic polio (VAPP) at historical rates of approximately 1 case per 2.5 million doses distributed in the United States [10] and 2.9-4.7 cases per million births worldwide in OPV-using countries, giving an estimated global burden of about 300-500 cases per year [11]. In addition, as a small mRNA virus, OPV can lose its attenuating mutations and vaccine-related viruses revert back toward WPV to become a circulating vaccine-derived poliovirus (cVDPV), which occurs in the context of prolonged circulation in populations with high susceptibility and can lead to outbreaks [12]. Moreover, in a very small number of individuals with B-cell immunodeficiencies, receiving OPV can lead to prolonged or chronic infections and the development of immune deficient vaccine-derived polioviruses (iVDPVs) [13]. Given these complications, the GPEI expects to coordinate the cessation of OPV use after global eradication of WPVs to minimize the risks associated with live polioviruses (LPVs, i.e., WPVs, VDPVs, OPVs, and OPV-related viruses) [4,14]. At that time, inactivated poliovirus vaccine (IPV) [15] will represent the only vaccine option [16]. However, OPV cessation of any given serotype cannot safely occur until assurance of global interruption of transmission of that serotype. Because the majority of poliovirus infections occur asymptomatically, the possibility of silent circulation complicates the confirmation of local and global interruption of WPV or cVDPV transmission.

A previous statistical analysis [17] and a mathematical infection transmission model of a simple and small hypothetical population [18] that assumed perfect surveillance suggested that it takes approximately 3-4 years without WPV-caused paralytic cases to achieve 95% confidence about the interruption of transmission. A reanalysis of one of these studies [18] demonstrated how varying assumptions that reflect actual conditions about population immunity, vaccination strategies, and seasonal fluctuations in transmissibility impact estimates of the probability and duration of silent circulation [19]. The reanalysis also identified the need to consider the role of previously-vaccinated or infected individuals (i.e., partially infectible individuals) who remain immune to paralytic disease, but not to reinfection, and their potential participation in silent transmission of the virus [19,20]. The reanalysis confirmed the importance of the paralysis-to-infection ratio (PIR) as a key input, [18,19] which suggests the need to perform separate analyses for each serotype. While the reanalysis provided several insights relevant to current conditions, it relied on a hypothetical population, the use of a highly-simplified transmission model that included either OPV or IPV, and it did not account for imperfect surveillance [19]. In this paper, we seek to characterize the confidence about no circulation as a function of time without detected events (i.e., cases or positive sewage samples) in populations with relatively recent circulation. We model populations that may represent conditions like the actual last global reservoirs of WPV transmission and the actual environmental WPV detection that occurred in Israel in 2013. Prior to discussing the model and methods, the next section provides an overview of the different components of polio surveillance, which provides important context about the types, quality, and timing of information available.

### Poliovirus surveillance

**Figure 1:**
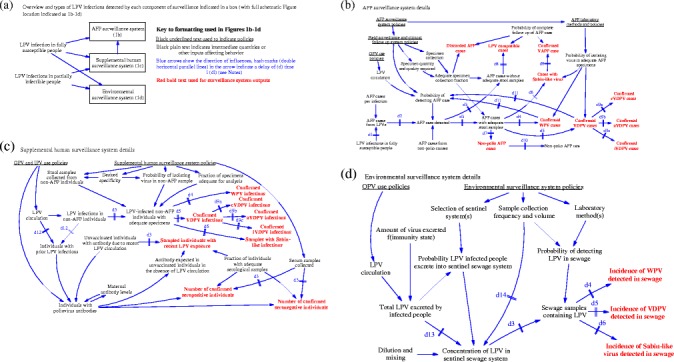
**Schematic of poliovirus surveillance. (a)**. Overview and sources of LPV infections detected by each component of surveillance indicated in a box (with full schematic Figure location indicated as 1b-1d). **(b)**. AFP surveillance system details. **(c)**. Supplemental human surveillance system details. **(d)**. Environmental surveillance system details. Notes: blue arrows show the direction of the influence; hash marks (double horizontal parallel lines) in the arrow indicate a delay of (d) time i (di); d1, time from onset of infection to onset of paralysis (incubation period); d2, time to detect AFP case in the field; d3, time to collect sample and ship sample to laboratory; d4,time to confirm WPV in specimen; d5, time to confirm VDPV in specimen; d6, time to confirm Sabin-like virus in specimen; d7, time to confirm the absence ofpoliovirus in specimen; d8, time of clinical follow-up; d9, time of VDPV follow-up investigation (varies such that d9a < d9b << d9c); d10, time for non-polioAFP reporting and averaging; d11, time of effective surveillance intensity ramp-up (if confirmed cases increase) or decrease (in the absence of confirmed cases);d12, time to recover from infection; d13; dilution time; d14, time between sample collection; APF, acute flaccid paralysis; IPV, inactivated poliovirus vaccine;LPV, live poliovirus; OPV, oral poliovirus vaccine; VDPV, vaccine-derived poliovirus (preceded by “c” to specify circulating VDPV, “i” to specify virus froman immunocompromised long-term excretor, or “a” to indicate an ambiguous source (i.e., not classifiable as “c” or “i”); WPV, wild poliovirus

Fully susceptible individuals (i.e., those never infected with LPVs or never successfully IPV-vaccinated) can become paralyzed following infection with a LPV and present with acute flaccid paralysis (AFP) caused by polio. In contrast, partially infectible individuals (i.e., those who acquired immunity from prior LPV infections or successful IPV doses) benefit from complete, permanent protection from paralytic poliomyelitis; however, we call them “partially infectible” to indicate that they can become re-infected and participate to some degree in poliovirus transmission (depending on their immunity state and extent of mucosal immunity waning). Figure 1 shows an overview (Figure 1a) and the various components (Figure 1b-d) of poliovirus surveillance. Poliovirus surveillance can include efforts to detect AFP cases and analyze their stool samples to look for poliovirus (i.e., the AFP surveillance system shown in the top box in Figure 1a with the details given in Figure 1b), efforts to collect biological samples (e.g., stool, serum, oropharyngeal) from selected individuals not presenting with AFP to look for evidence of recent poliovirus infection (i.e., human supplemental surveillance shown in the middle box in Figure 1a with the details given shown in Figure 1c), or efforts to collect sewage samples to detect the presence of poliovirus excretion by individuals in the catchment area (i.e., environmental surveillance shown in the bottom box in Figure 1a with the details given in Figure 1d). We include a key to interpreting the information in Figure 1b-d to the right of the boxes with the various components in Figure 1a, and notes at the bottom that provide the details of abbreviations. Throughout the various poliovirus surveillance systems, delays exist that limit the ability to rapidly confirm the presence of a WPV or cVDPV. Figure 1 indicates delays in the system using hash marks on the blue arrows labeled with di, where i represents a number that corresponds to the specific delay defined in the notes at the bottom.

The Global Polio Laboratory Network (GPLN) represents one of the most sophisticated laboratory resources in the world, and it continues to develop technologies that improve overall surveillance quality and provide valuable information [21]. The GPLN primarily focuses on AFP surveillance, which analyzes stool samples from AFP cases detected through active field surveillance for the presence of poliovirus. The information from the AFP surveillance system depends on the field and laboratory policies and OPV use policies, which along with WPV circulation impact overall LPV circulation. The GPLN analyzes stool samples from detected AFP cases and provides numerous information outputs (i.e., shown using red bold text in Figure 1b), including the incidence of virologically-confirmed WPV and VDPV paralytic polio cases, AFP cases with Sabin-like (i.e., closely-related to OPV) poliovirus, polio-compatible cases (i.e., clinically compatible with poliomyelitis, but no adequate specimens available to virologically-confirm or disconfirm polio), and non-polio AFP cases (Figure 1b) [22]. A determination of “adequate” for samples from an AFP case requires obtaining “two stool specimens of sufficient quantity for laboratory analysis, collected at least 24 hours apart, within 14 days after the onset of paralysis, and arriving in the laboratory by reverse cold chain and with proper documentation [23].” Isolation of a VDPV case triggers further investigation, which then identifies the case as a confirmed cVDPV case, a confirmed immunodeficient prolonged or chronic excretor (i.e., iVDPV) case, or an isolated case with an ambiguous source (i.e., aVDPV case). Isolation of a Sabin-like virus from an AFP case after epidemiological and clinical follow-up and expert review can lead to designation of the case as a confirmed VAPP case. Several factors affect the overall efficiency and reliability of the AFP surveillance system, [24] including the probability of detecting AFP cases in the field, which depends on the rate at which the system detects the “background” occurrence of non-polio AFP cases, timeliness, quantity and quality of specimen collection, proportion of cases with inadequate specimens receiving clinical follow-up, and the laboratory sensitivity of detecting a virus from an LPV-infected AFP case with adequate specimens. Significant variability may exist within the system (e.g., the laboratory confirmation process in sub-Saharan Africa and Asia ranged from 4 to 10 weeks in 2003-06 [25]). Extensive performance criteria and active quality control monitoring for the GPLN and AFP surveillance system [22] require that the annual AFP rate should exceed 1/100,000 people under 15 years of age in the catchment area [26]. The GPLN also requires the investigation of at least 80% of cases, which must occur within 48 hours of notification and include the collection of 2 adequate stool specimens obtained 24-48 hours apart within 14 days of the onset of paralysis [26]. In some places, surveillance includes the collection of adequate stool specimens from 5 or more neighborhood contacts of cases with inadequate stool specimens [27]. Higher non-polio AFP rates in any given population indicate a lower probability of missing clinical cases (i.e., higher sensitivity). However, heterogeneity of the non-polio AFP rate may imply effectively lowered sensitivity if low non-polio AFP rates correlate with the places most likely to incur polio cases.

Supplemental human surveillance activities, which include stool sampling from individuals without paralytic symptoms but with elevated probability of poliovirus excretion (e.g., contacts of AFP cases, meningitis cases) and/or serologic surveys, may also provide poliovirus surveillance information as shown in Figure 1c. High non-polio AFP rates (e.g., exceeding 2/100,000) may indicate effectively expanded case definitions and imply a higher probability of randomly detecting polioviruses that did not cause paralysis (i.e., lower specificity). Serological surveillance can detect evidence of a history of any poliovirus exposure, including any LPV infection and vaccination with OPV or IPV, but it cannot identify the virus or time of the exposure, and thus it currently cannot reliably detect circulation as it occurs. However, after OPV cessation, serological surveillance or targeted stool surveys may offer the ability to detect evidence of recent or ongoing LPV circulation, and consequently we include Figure 1c for completeness.

Figure 1d provides a schematic for environmental surveillance, which can offer a more sensitive signal than AFP surveillance, particularly in communities with no clinical cases of polio [23,28-33]. The use of OPV complicates environmental surveillance, because widespread OPV infections imply that many samples will contain LPVs (mostly Sabin-like viruses), indicating the need for additional processing to screen out OPV-related viruses. However, studies demonstrate the utility of environmental surveillance with respect to measuring the length of poliovirus persistence in the environment after OPV vaccination campaigns and after a national switch from OPV to IPV for RI. Many countries currently use environmental surveillance routinely (e.g., Afghanistan, China, Egypt, India, Israel, Japan, New Zealand, Nigeria, Pakistan, Russia and some former Soviet states, several European countries) although the systems differ considerably [34]. Significant advantages of environmental surveillance include the potential to detect silent circulation of WPVs and cVDPVs before any AFP cases occur (e.g., Egypt, [35] India, [36] Israel [37]). Experience in India demonstrates that careful selection of sampling sites with open sewers in highly-populated or high-risk areas can overcome the limitation of inadequate sewage networks [36]. The value of environmental surveillance with respect to early detection and the opportunity to prevent any cases using vaccination became clear in early 2013 when Israel detected the circulation of WPV1 in the absence of any evidence of clinical cases [37].

All of the factors that influence poliovirus surveillance sensitivity for all components may change over time. For example, after the detection of the first case in an outbreak, sensitivity of detection of subsequent cases typically increases due to intensification of all aspects of surveillance. Similarly, surveillance sensitivity may decrease with time after the last detected case. Detection of a WPV in a population depends on the probability of detecting at least one infected person from a population with WPV circulating, [38,39] but although the case may not necessarily occur in that population (e.g., on-going circulation of WPV in Sudan following isolation of a positive sewage sample taken in Egypt). Despite the extensive efforts to ensure high-quality surveillance information globally, gaps in surveillance still exist, particularly in conflict areas with access limitations and in resource-poor areas [40]. This suggests that the quality of surveillance represents an important consideration in the context of certifying an area as free of WPVs (or more broadly free of LPVs). Overall poliovirus surveillance provides valuable, but imperfect information, and this motivates the need to consider the impacts of imperfect information in the polio end game.

The components of the poliovirus surveillance system result in population-specific probabilities of detection and confirmation of the presence of poliovirus circulation, which we consider as we model the confidence about no circulation as a function of time since the last detected event. Specifically, the model described in the next section considers a probability of detecting and confirming any individual paralytic case from AFP surveillance and a probability of detecting and confirming the presence of poliovirus in sewage from environmental surveillance for each population including that type of surveillance. While numerically modeling each surveillance system in each specific population requires more data and remains beyond the scope of this work, we found that visualizing the relationships between the various components helped to convey the nature of the information provided and may help inform national and global choices that will influence overall surveillance quality and timeliness.

## Methods

We build on our understanding of poliovirus surveillance and prior comprehensive poliovirus infection transmission models [7,20,41-43] to estimate the confidence about (probability of) no circulation in each modeled population as a function of the time since the last detected event. We model populations with relatively recent WPV transmission (both endemic and imported) that rely on AFP surveillance and the actual environmental surveillance detection of WPV serotype 1 (WPV1) that occurred in Israel in 2013 [44]. Although Nigeria started some environmental surveillance efforts in 2011, the scale of these efforts until recently remained relatively small, and consequently we do not consider the potential impact of this environmental surveillance. We recognize that only increasingly localized and shrinking geographic areas in three countries (i.e., Afghanistan, Nigeria, and Pakistan) maintain indigenous WPV1 transmission, but importations into previously WPV-free countries continue to pose a threat of re-established transmission and potential silent circulation, [1] and the emergence of cVDPVs pose a real threat [12]. Consequently, we focus on identifying some scenarios that reasonably represent different types of areas and conditions that warrant modeling because of their population immunity, characteristics, and/or surveillance quality. Similar to prior studies [18,19] this analysis focuses on the detection within a closed population in which an LPV might circulate silently (i.e., without detection).

The model divides the population according to immunity states, including fully susceptible individuals (i.e., those never infected with LPVs or successfully IPV-vaccinated), maternally immune individuals (i.e., children born to mothers with any active recent or historic immunity), and partially infectible individuals (i.e., those who acquired immunity from 1, 2, or 3 or more prior successful IPV doses, or from 1 or 2 or more prior LPV infections) [7]. As noted in the prior section, partially infectible individuals benefit from complete, permanent protection from paralysis; however, re-infection can occur and lead to different degrees of participation in poliovirus transmission (depending on their immunity state and extent of mucosal immunity waning). Only fully susceptible and maternally immune individuals with insufficient maternal antibodies remaining to provide protection can become paralyzed when infected with a LPV for the first time. The model includes a 6-stage infection process, a 5-stage immunity waning process, and a 20-stage poliovirus reversion process for both fecal-oral and oropharyngeal routes of transmission. We divide the population into a number of age groups that varies depending on the modeled situation. However, regardless of the scenario settings we combine the age groups into 3 preferentially mixing age groups (i.e. 0-4, 5-14, and 15 or more years) [7,20,41-43]. We characterize population immunity to poliovirus transmission by computing the age-and-sub-population-mixing-adjusted effective immune proportion (EIPM) [42]. We also define an immunity threshold, EIP*, which depends on the basic reproductive number for the population and virus serotype (R_0_), with EIP* = (1- 1/R_0_). For EIPM > EIP* infections eventually die out [42]. We use the EIPM and R_0_ to estimate the net reproductive number (Rn), defined as (1-EIPM) × R_0_, which represents the average number of secondary transmissions generated by a single infection given the population immunity at the time Rn has a threshold of 1 regardless of the assumed R_0_ for a population and serotype. Thus, for Rn > 1 (i.e., EIPM < EIP*) continued transmission can occur, while for Rn < 1 transmission will die out (i.e., EIPM > EIP*). We model 4 populations (i.e., qualitatively different conditions related to the transmission potential, expected population immunity at the time of WPV1 or WPV3 certification, OPV take rates, and surveillance quality) as shown in Table 1 [41-44]. For each population, we specify demographic information, poliovirus transmissibility and seasonality, and the history of live poliovirus exposure, including OPV use for RI or SIAs. The Additional file 1 provides details about the model, including updates for recent immunization activities and assumptions used to model die-out and characterize population immunity. We run the existing deterministic poliovirus transmission model for each situation and serotype up to a point in time shortly before die-out occurs (Table 1) and then transform it to a discrete, stochastic model that we run 1,000 times (see Additional file 1). For the situation representing Israel, we focus on iterations consistent with the outbreak response that actually occurred (i.e., we only allow the iterations with one or fewer paralytic cases occurring after mid-July 2013, because the occurrence of actual cases prior to that date would have likely changed the outbreak response).

**Table 1:** **List of scenario-specific model inputs**

***Model input***	**Scenario 1 (Northern India)**	**Scenario 2 (Northwest Nigeria)**	**Scenario 3 (Tajikistan)**	**Scenario 4 (Israel)**
Population [model references]	Bihar and Western Uttar Pradesh (WUP), each state with 8 age groups and a preferentially-mixing under-vaccinated subpopulation [7,41,43]	7 states combined into one population with 11 age-groups with an under-vaccinated subpopulation [7,42,43]**	3 administrative regions primarily affected by the 2010 outbreak with 11 age groups [7,43]	National population with 13 age groups divided into two preferentially-mixing geographical regions that each include a preferentially-mixing under-vaccinated subpopulation [44]
Qualitative characteristics				
Overall transmission potential	High	Medium	Medium	Low
Population immunity	High	Medium	Medium	Medium
OPV take rate	Low	Medium	Medium	High
Surveillance quality	High	Medium	Low	High
Stochastic model start dates				
Serotype 1	2008	2013	2009	2013
Serotype 2	2008	2013	-	-
Serotype 3	2008	2010	-	-
Average R_0_ (WPV1)	13	7.5	8	5
Average EIP* (WPV1)	0.92	0.87	0.88	0.80
SIA coverage (general population)	Time series (Bihar: 0.86-0.95; WUP: 0.84-0.94)	0.85	0.95	0.80
Average RI coverage	0.65	0.11	0.40	0.95
AFP surveillance p = (p_1_,p_2_,…, p_i_)	General (0.95, 0.95, …, 0.95) Subpopulation (0.8, 0.8, …, 0.8)	General (0.80, 0.85, 0.90, …, 0.90)	General (0.20, 0.25, 0.50, 0.50, 0.73, …, 0.73)	General (0.50, 0.75, 0.95, …, 0.95)
		Subpopulation (0.54, 0.57, 0.60, …, 0.60)		
Environmental surveillance				
s = (s_1_,s_2_)	-	-	-	(0.30, 0.90)
Threshold (EI*)	-	-	-	South: (60,60,60,60,60,12,12,10,13,20,20,20,20,20,20)
				Rest of Israel: (113,136,227,170,97,32,43,15,62,68,68,136,38,40,34)

*Abbreviations:* EIP*, threshold of effective immune proportion; R_0_, basic reproductive number; WPV1, wild poliovirus serotype 1

** Includes updated SIAs rounds in September 2013 and March 2014 from tOPV to bOPV; updated the current path scenario with 2 rounds using tOPV (in August and November) for 2014 and 3 annual rounds using tOPV (in March, August and November) from 2015 forward until OPV2 cessation; updated RI coverage for consistency with the 2013 DHS survey, [45] with relative RI coverage in the subpopulation of 20% in 2012, 25% in 2013 and 30% in 2014 and forward.

We synthesize the following results, similar to prior analyses [18,19]:

- POE - the probability of eradication defined as the fraction of stochastic iterations in which die-out occurs (i.e., prevalence below the transmission threshold)
- DEFP - the detected-event-free period defined as the time in months since the last detected case (AFP) or positive isolate (environmental surveillance)
- CNC - confidence about no circulation given the DEFP approximated as (1 - the number of DEFPs equal to t months with ongoing WPV circulation, divided by all DEFPs of t months)
- CNCx% - the time when the confidence about no circulation exceeds x% (i.e., CNC95%, CNC99%)
- TUC - the time of undetected circulation after the last detected-event (for those iterations in which extinction occurs)
- TUCx% - The x^th^ percentile of the TUC (i.e., TUC95%, TUC99%).

For this analysis, we focus on detected-events (i.e., detecting cases from AFP surveillance and positive isolates that indicated the presence of infections from environmental surveillance) and the DEFP instead of the case-free period used previously [18,19] to account for the different information provided by the two components of poliovirus surveillance used. To emphasize our focus on no circulation in the absence of cases and in the context of less than perfect surveillance, we use the time of undetected circulation (TUC) rather than the time of silent circulation (TSC) assuming perfect surveillance used previously (i.e., TUC with perfect surveillance equals TSC) [18,19].

We explicitly consider the impact of imperfect information from surveillance and provide a comparison to perfect information as a point of reference. For each modeled situation, we define a detection function (DF) as an indicator of overall surveillance quality and to account for imperfect surveillance. The detection function describes the probability of detecting the i^th^ case (p_i_, used for AFP surveillance) or i^th^ sewage sample that contains poliovirus (s_i_, used for environmental surveillance) in a cluster. We define a cluster as a series of sequential detected cases or sequential virus detections in sewage in a given geographical subpopulation, with no more than one year between successive detections. For environmental surveillance, detection depends on the number of individuals excreting poliovirus into the sewage system in a geographical subpopulation. We define the effective infectiousness-weighted number of infectious individuals (EI) and compare this to the threshold (EI*) required for the system to detect the virus in the sewage. We define s_i_ as the probability of detecting poliovirus in the i^th^ sewage sample given that EI > EI*. Thus, each time when sample collection should occur (i.e., every month) we check whether the weighted number of excreting individuals exceeds the EI*.

We characterize the different inputs for surveillance quality assuming variable levels of quality of the (sub)national surveillance systems, with better systems receiving relatively higher values for all p_i_. This framing allows for a relatively higher probability of missing the first case, with decreasing probabilities of missing subsequent cases in the same temporal cluster for the fixed spatial area, depending on the situation-specific assumptions (Table 1, details on methods and assumptions provided in the Additional file 1).

## Results

**Figure 2:**
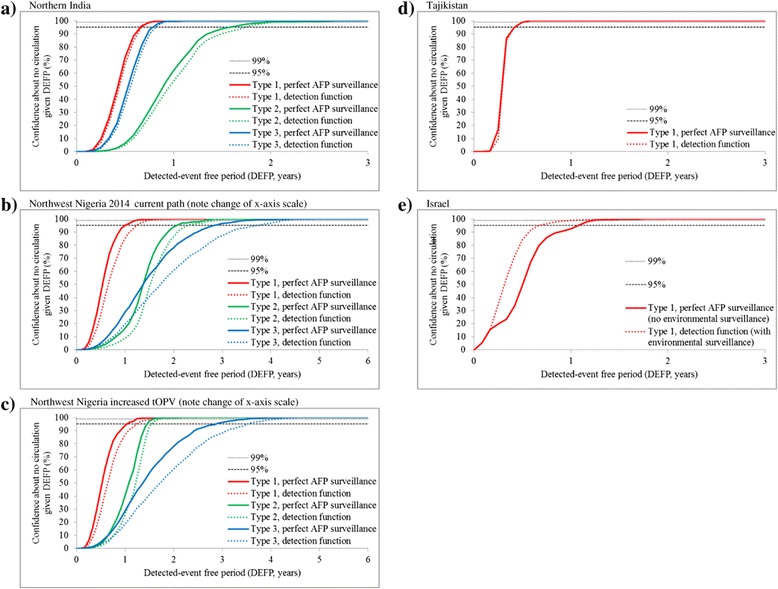
**Confidence about no circulation as a function of the detected-event free period and lines provided to indicate 95% and 99% for reference. a)**. Northern India. **b)**. Northwest Nigeria 2014 current path (note change of x-axis scale). **c)**. Northwest Nigeria increased tOPV (note change of x-axis scale). **d)**. Tajikistan. **e)**. Israel.

Figure 2 presents the confidence about no circulation as a function of time without detected events, with black horizontal lines at the top showing the 99% (small dots) and 95% (larger dots) levels for reference. Each solid line (red, green, blue) represents the result using perfect AFP surveillance for the indicated serotype. Each dotted line of the corresponding color presents the impact of imperfect AFP surveillance (Figure 2a-d) or environmental surveillance (Figure 2e), as characterized for the different detection functions in Table 1.

**Figure 3:**
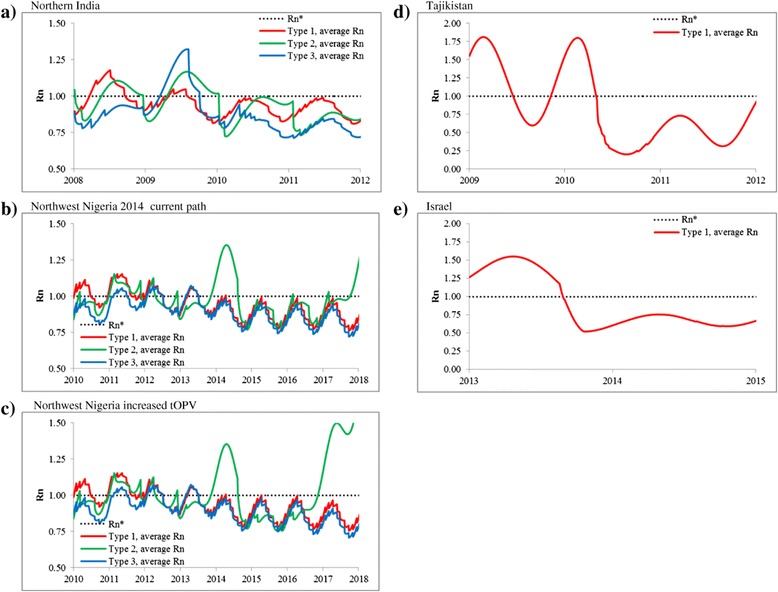
Net reproductive number for each scenario relative to the threshold for sustained transmission (Rn* = 1) for the relevant time period modeled. a) Northern India. **b)** Northwest Nigeria 2014 current path. **c)** Northwest Nigeria increased tOPV. **d)** Tajikistan. **e)** Israel.

Figure 3 further demonstrates the importance of the relationship between population immunity to transmission and undetected circulation using Rn (the Additional file 1 shows the corresponding figures for EIPM, which we use to characterize population immunity for reference).

Table 2 reports the CNC95%, CNC99%, TUC95% and TUC99% for the situations assuming perfect AFP surveillance only (top) and with our best estimates of actual, imperfect surveillance quality (bottom).

**Table 2:** **Expected detected-event free period (DEFP) required for 95% and 99% confidence about no circulation (CNCx%) and time of undetected circulation between the last paralytic case and die-out (TUCx%) by serotype (based on 1,000 iterations) assuming perfect surveillance (top) and imperfect surveillance (bottom)**

**Virus**	**WPV1 (** ***PIR*** **= 1/200)**				**cVDPV2 (** ***PIR*** **= 1/ 2000)**				**WPV3 (** ***PIR*** **= 1/1000)**			
**Metric**	**CNCx%**		**TUCx%**		**CNCx%**		**TUCx%**		**CNCx%**		**TUCx%**	
**x%**	**95%**	**99%**	**95%**	**99%**	**95%**	**99%**	**95%**	**99%**	**95%**	**99%**	**95%**	**99%**
Population	**DEFP values assuming perfect AFP surveillance without environmental surveillance**											
Northern India	0.67	0.83	0.65	0.74	1.58	2.00	1.39	1.96	0.83	0.92	0.77	0.86
Northwest Nigeria												
- current path	1.00	1.25	0.98	1.23	2.08	2.67	1.95	2.28	2.83	3.58	2.61	3.53
- increased tOPV	1.08	1.25	1.01	1.24	1.50	1.67	1.36	1.58	2.92	3.58	2.65	3.53
Tajikistan	0.42	0.50	0.35	0.38	-	-	-	-	-	-	-	-
Israel	1.25	1.42	1.19	1.36	-	-	-	-	-	-	-	-
Population	**DEFP values assuming AFP surveillance with quality based on Table** 1 **(including environmental surveillance in Israel)**											
Northern India	0.67	0.83	0.66	0.77	1.75	2.25	1.51	2.19	0.83	0.92	0.80	0.87
Northwest Nigeria												
- current path	1.25	1.50	1.22	1.48	2.33	2.92	2.16	2.53	3.75	4.33	3.62	4.27
- increased tOPV	1.25	1.58	1.22	1.54	1.58	1.75	1.47	1.66	3.58	4.17	3.45	4.17
Tajikistan	0.42	0.50	0.36	0.38	-	-	-	-	-	-	-	-
Israel	0.92	1.17	0.76	1.15	-	-	-	-	-	-	-	-

*Abbreviations:* CNCx%, DEFP at which the confidence about no circulation exceeds x%; cVDPV, circulating vaccine-derived poliovirus; DEFP, detected-event-free period; OPV, oral poliovirus vaccine; PIR, paralysis-to-infection ratio; tOPV, trivalent OPV; TUCx%, time at which the probability of undetected WPV circulation after the true last case becomes exceeds x%; WPV, wild poliovirus.

Figure 2a confirms that differences in the PIRs significantly affect the length of time required to observe paralytic cases, with lower values (serotype 1 > serotypetype 3 > serotypetype 2) indicating the need to wait longer because cases occur less frequently per infection [19]. Figure 3a for northern India shows Rn consistently below 1 for all three serotypes after 2010. In Figures 2b and 2c, the curves for serotype 3 cross the curves for serotype 2. This occurs because in the Nigeria model, gaps in vaccination in the sub-population in 2010-2013 gave a long tail of serotype 3 circulation with no cases prior to die out. Given that our model assumes a rapid ramp-up in population immunity and high surveillance quality for northern India but not for northwest Nigeria (Table 1), [41,42] the contrast between Figures 2a and 2b shows that high population immunity (compare Figures 3a and 3b) and high AFP surveillance levels (Table 2) lead to significantly shorter CNCs and TUCs. Figure 3b shows a clear risk of transmission for serotype 2 for northwest Nigeria, with the population immunity for Nigeria for serotype 2 continuing to hover around the threshold for the current path strategy for most stochastic iterations (Figure 2b). The alternative increased tOPV scenario with 4 instead of 3 tOPV rounds in 2015 allows for the earlier elimination of cVDPV2 in all stochastic iterations (Figure 3c), which would support the global coordination of OPV2 cessation in 2016 and further demonstrates the importance of high population immunity for reducing the duration of potential undetected circulation. In contrast, continuing only 2 annual tOPV rounds per year from 2014 forward causes population immunity to hover around the threshold, with very long times between cases and no elimination of cVDPV2 transmission (not shown). Figures 3b and 3c also show the relatively rapid loss of population immunity (i.e., increase in Rn) for serotype 2 that occurs after OPV2 cessation (i.e., in 2016 for Figure 3c and 2017 for Figure 3b).

Figures 2d and 2e represent importations into previously polio-free areas. Figure 2d shows an outbreak in which many cases occurred relatively quickly leading to relatively short intervals between cases and motivating rapid and intensified outbreak response activities. The outbreak response resulted in short TUCs, with 95% chance of interruption of transmission within 4 months of the last detected case (Table 2) and correspondingly short potential undetected circulation (i.e., short CNC95% and CNC99%). In other polio-free countries with no recent polio cases, surveillance similarly may not perform well initially, but the dynamics of the outbreak and response may differ in other situations, leading to different outbreak kinetics and CNCs.

Figure 2e shows a situation of WPV1 transmission detected by environmental surveillance in the absence of any detected paralytic cases. The solid curve in Figure 2e appears less smooth compared to some of the other curves in Figure 2. In approximately 50% of the stochastic iterations the virus dies out after only a short period of circulation (i.e., it does not “take off”), implying an exponential decrease in the probability of undetected circulation during the first months that corresponds to the exponential process of recovery of the initial infections. In approximately 25% of the iterations 1 stochastic case occurs (i.e., shortly before or sometime after the outbreak response), which implies less than approximately 1 year between cases. In another approximately 25% of iterations, no cases occur despite circulation of virus for approximately 1 year. Figure 2e and Table 2 suggest that high-performing environmental surveillance could reduce the CNC95% by 26%, depending on the frequency and effectiveness of environmental sampling.

Depending on the situation-specific characteristics (e.g., the overall population immunity, endemic versus outbreak conditions, and virus serotype), the model suggests time periods of 0.5 to 3 years without circulating paralytic cases caused by WPV or cVDPV required to achieve 95% confidence in the interruption of transmission in the context of perfect AFP surveillance (Table 2). This interval could become longer if AFP surveillance becomes less than perfect (dotted lines in Figures 2a, 2b, 2c, and 2d) or shorter using sensitive environmental surveillance (dotted line in Figure 2e).

## Discussion

Current policies require a period of at least 3 years of no paralytic polio cases detected by the AFP surveillance system to certify an area as free of WPV. Our analysis suggests that specific situations and serotypes will differ such that 3 years may imply more or less than 95% confidence and demonstrating that the quality of the information impacts the time. Policy makers should recognize that choices made to achieve and maintain high levels of population immunity could impact the success or failure of polio eradication and the investment required in ongoing surveillance to ensure confidence about the absence of silent circulation when observed cases and/or signals from environmental surveillance stop [46,47]. Correlation between the populations at greatest risk of undetected circulation and poor surveillance quality suggest the need to specifically focus on underserved areas. Recognizing these areas may motivate the exploration of the development of environmental surveillance systems that could potentially reduce the time required to reach a high level of confidence about the absence of circulation in these areas, which will likely drive global decision making. Considerable variation in poliovirus surveillance activities (and the associated population-specific values for the components in Figures 1b-1d) leads to different degrees of quality, and policy makers will need to consider the impacts of imperfect information.

Early detection of circulating infections provides an important opportunity to increase population immunity, decrease Rn, and prevent any cases from occurring. In Israel, population immunity fell below the threshold and transmission occurred, but this did not lead to cases due to the protection from paralysis provided by high coverage with IPV. The ability of high coverage with IPV to prevent cases but potentially not prevent or disrupt transmission in some situations may decrease the ability to detect transmission using AFP surveillance.

Serotype differences in the PIRs imply significant differences between the relevant DEFP values, which may influence OPV cessation policy development. For example, the relatively low PIR of cVDPV2, if combined with insufficient population immunity against serotype 2 and heterogeneity in surveillance quality in regions like northwest Nigeria, may lead to significant increases in the DEFP values, which would indicate the need for longer periods of time required without detected events to certify the interruption of LPV transmission. Some countries will need to increase population immunity prior to OPV cessation to safely stop LPV transmission and rapidly obtain confidence about the absence of transmission.

Our analysis remains limited by the model assumptions, including assumptions made to translate the differential-equation based model into a stochastic model related to use of the transmission threshold. For example, using the criterion for die-out in this analysis represents prevalence below the transmission threshold rather than absolute 0 total infected individuals. Nonetheless, we believe the analysis provides useful insights relevant to the polio endgame.

As we consider the prerequisites for OPV cessation and approach key decision points, countries will need to recognize that increasing population immunity and the actual quality of surveillance will impact the risks of undetected circulation and the confidence associated with different time periods of no observed cases. The potential degradation of surveillance quality suggests the need to ensure a commitment to high-quality surveillance and/or requires a longer time of no detected events. Environmental surveillance may provide an important opportunity to reduce the time required to feel confident about the absence of undetected circulation, although the value of the information provided will depend on the system design.

## Additional file

Additional file 1:
**Technical appendix.**

## Abbreviations

AFP: Acute flaccid paralysis
bOPV: Bivalent OPV (serotypes 1 and 3)
CNC: Confidence about no circulation given the DEFP
CNCx%: Confidence about no circulation exceeds x% given the DEFP
d: Delay of
di: Delay of time i (used to distinguish types of delays that occur in various parts of polio surveillance)
d1: Time from onset of infection to onset of paralysis (incubation period)
d2: Time to detect AFP case in the field
d3: Time to collect sample and ship sample to laboratory
d4: Time to confirm WPV in specimen
d5: Time to confirm VDPV in specimen
d6: Time to confirm Sabin-like virus in specimen
d7: Time to confirm the absence of poliovirus in specimen
d8: Time of clinical follow-up
d9: Time of VDPV follow-up investigation
d10: Time of non-polio AFP reporting and averaging delay
d11: Time of effective surveillance intensity ramp-up (if confirmed cases increase) or decrease (in the absence of confirmed cases)
d12: Time to recover from infection
d13: Dilution time
d14: Time between sample collection
DEFP: Detected-event-free period
DF: Detection function
EI: The minimal number of effective infectiousness-weighted individuals who are infectious
EI*: The threshold required to detect the virus in the sewage
EIP*: threshold effective immune proportion
EIPM: age-and-sub-population-mixing-adjusted effective immune proportion above which infections eventually die out
GPEI: Global Polio Eradication Initiative
GPLN: Global Polio Laboratory Network
IPV: Inactivated poliovirus vaccine
LPV: Live poliovirus
OPV: Oral poliovirus vaccine
p_i_: Probability of detecting the i^th^ case in a cluster of cases
PIR: Paralysis-to-infection ratio
POE: Probability of extinction
R_0_: Basic reproductive number
RI: Routine immunization
Rn: Net reproductive number
s_i_: The probability of detecting poliovirus in the i^th^ sewage sample in a cluster of environmental detections
SIA: Supplemental immunization activity
tOPV: Trivalent OPV
TUC: Time of undetected circulation between the last paralytic case and WPV interruption
TUCx%: Time at which the probability of undetected WPV circulation after the true last case exceeds x%
VAPP: Vaccine associated paralytic polio
VDPV: Vaccine-derived poliovirus (preceded by “c” to specify circulating VDPV, “i” to specify VDPV virus from an immunocompromised long-term excretor, or “a” to indicate an ambiguous VDPV source (i.e., not classifiable as “c” or “i”)
WPV: Wild poliovirus
