# Supplementary material for: Modeling undetected live poliovirus circulation after apparent interruption of transmission: implications for surveillance and vaccination
Source: BMC Infect Dis. 2015 Feb 18;15:66. doi: 10.1186/s12879-015-0791-5 (PMC4344758; doi:10.1186/s12879-015-0791-5)
Supplement: Additional file 1: — Technical appendix. [file 12879_2015_791_MOESM1_ESM.docx]

**Additional file 1**

**A1: Model details related to immunization inputs and viral die-out**

We characterize the impact of each SIA by its true coverage and its repeated missed probability among individuals targeted in the preceding SIA round,[43] and we model the cumulative effect of IPV doses administered as part of the RI schedule as described elsewhere.[41] We further use a transmission threshold for the effective proportion infectious (i.e., the infectiousness-weighted number of infectious individuals in any mixing group divided by the total number of people in the mixing group) below which we set the force-of-infection of any given LPV strain to 0. We calibrated this threshold so that it produces both realistic times of die-out of LPVs and realistic OPV evolution and emergence characteristics for cVDPVs across a range of situations in the deterministic differential-equation based poliovirus transmission model.[7, 41-43] This threshold simulates the reality that very low levels of poliovirus prevalence in a population do not sustain transmission due to local heterogeneity and stochastic die-out. Given that we do not characterize local heterogeneity or die-out and we maintain the OPV evolution process as characterized in the differential equation based model, we continue to use the transmission threshold for the stochastic model. Thus, the criterion for die-out in this stochastic model analysis represents prevalence below the transmission threshold rather than recovery from infection of the last infectious individual.

For northwest Nigeria, we updated the model to reflect actual vaccine use for SIAs that occurred prior to September 2014 (instead of the then-planned SIAs used in the prior analysis[1]). Specifically, compared to the prior analysis, we changed the rounds for September 2013 and March 2014 from tOPV to bOPV, because Nigeria actually used bOPV for those rounds. We updated the current path scenario to assume that Nigeria would conduct 2 rounds using tOPV (in August and November) in 2014 and 3 annual rounds using tOPV (in March, August and November) from 2015 forward until OPV2 cessation. We accounted for the current global policy of planned coordinated OPV2 cessation by changing all RI and SIAs to bOPV after the assumed global tOPV-bOPV switch date. In the context of updating the model to reflect the situation as it evolved since our prior analysis,[1] we further adjusted the relative SIA coverage in the under-vaccinated subpopulation for the September and October 2013 SIAs (i.e., prior to the observed uptick in cVDPV2 cases) to 0.15 with all other rounds from late 2013 on assuming relative SIA coverage of 0.2 as before.[1] Partly offsetting the reduction of tOPV use, we assume that efforts to identify more underserved communities led to some increase in RI use in the under-vaccinated subpopulation from 0 before 2012 to a relative coverage compared to the subpopulation with each of the 4 OPV doses in the RI schedule of 0.20% in 2012, 0.25% in 2013, and 0.30% from 2014 forward. We also increased the RI coverage for the general population starting in 2012 based on a recent study from 11.2% to 26.4% for the birth dose and from 11.6% to 13.9% for the third OPV dose, with partial coverage with 1 or 2 doses non-birth doses similarly updated to reflect the most recent data.[2] Despite the increased RI coverage and 3 annual tOPV rounds from 2015 forward, in the deterministic model, the current path scenario does not lead to cVPDV2 elimination in time to support a policy of globally-coordinated OPV2 cessation in April 2016 (i.e., die out by April 2015). Consequently, for the current path scenario, we assume OPV2 cessation would occur in April 2017. Given a global target to coordinate OPV2 cessation in April 2016, we modeled an increased tOPV scenario in which northwest Nigeria uses tOPV for one currently planned bOPV round (in January 2015) and thus maintains 4 tOPV rounds annually (January, March, August, and November) until coordinated global OPV2 cessation in April 2016. Although northwest Nigeria may introduce some use of IPV in 2014, we did not include it in the model in the absence of details about the specific use, presumed limited scale of IPV use, and an expected small projected impact of IPV on population immunity to transmission.[3, 4]

**A2: Description of stochastic model based on differential-equation based model**

To transform the deterministic differential-equation based model to a stochastic model, we first round the fractional number of individuals in each stock from the deterministic model at the time of the transformation to integer numbers. We do so by drawing random uniform numbers to determine the nearest integer below or above the fractional number from the deterministic model.

Each time step in one stochastic iteration starts with calculating all transition rates, which change the current state and a set of random draws for all calculated transition rates from the Poisson distribution with parameter equal to:

*transition rate* × *fixed time step*

with each random Poisson draw less than the size of the stock. Thus, the effective number of people that transition out of any given stock equals:

*min(randPoisson(transition rate(stock)* × *fixed time step), number of people(stock))*.

We choose a random number u ~ U [0; 1] to determine whether the event corresponding to 'infection' leads to a paralytic case. We assume a case occurred at time 0 and record the time since the last paralytic case on a monthly basis. We assume that stochastic iteration ends when virus is eliminated (i.e., the number of excreting individuals equals 0) or when the simulation year 2020. We use a fixed time step of 0.5 days.

We consider the probability of WPV circulation persisting given a DEFP of length t, by dividing the number of all DEFPs of length t months with WPV present by the total number of all DEFPs of length t months. We define the time of undetected circulation (TUC) after the last detected event (for those iterations in which extinction occurs) as the time between the last detected event and the end of WPV circulation. To approximate the probability of undetected infections as a function of time, the simulation may record multiple DEFP values between cases, because it evaluates the DEFP at each month by looking back from the current month to determine the number of months since the last case. Thus, in a given stochastic iteration if a DEFP value of 6 months gets recorded (i.e., at least 6 months occurred between 2 cases or after the last case), then this must follow recorded DEFP values of 5, 4, 3, 2, and 1. We can observe a TUC for each iteration of the simulation that ends with extinction. After creating the empirical cumulative distribution function (ECDF) of all of the TUC values (i.e., for all of the iterations in which extinction occurs), we can estimate the time after which the probability of occurrence of a longer TUC exceeds x% (TUCx%). Thus, the TUC95% represents the time t that satisfies ECDF(t) = P[TUC > t] >0.95.

By considering the fraction of DEFPs that occur in the context of WPV or cVDPV2 circulation, the CNCx% metric provides the confidence about no WPV or cVDPV2 circulation as a function of time without observed events. In contrast, the TUCx% metric tells us how long we might expect silent WPV or cVDPV2 circulation to continue after the last detected paralytic case if extinction occurs. We use the model to calculate Rn based on the average of 1,000 stochastic iterations minus any iterations that stopped due to prior die-out of transmission, which we compare to the dashed threshold line of Rn*=1.

**A3: Basis for assumptions about inputs for surveillance quality used to characterize the detection function (DF)**

As discussed in the main text, the DF for AFP surveillance depends on the number of recently detected paralytic cases. We define p_i_, as the probability of detecting the i^th^ case in a cluster of cases. We define a cluster as a series of sequential cases in a given geographical subpopulation, with each case occurring within a year of the previous case. We assume a “New Cluster” (NC) begins for any case that occurs 365 days after the last case that occurred within the geographical subpopulation. We define the “Case Detected Total” (CDTj) as a counter of the cases in cluster j and upon detection of the first case we define the case total in cluster 1 as 0 (i.e., CDT_1_=0). As each subsequent case occurs, we first check to see if it represents the first case of a new cluster (NC) for which we use p_1_ from the bottom of Table 1 to determine the probability of the surveillance system detecting the case, or if the case belongs to the current cluster, in which case we identify it as the i^th^ (i.e., the 2^nd^, 3^rd^, or a later) case in the current cluster and use the appropriate value for p_i_. Following detection of each case, we increment the appropriate CDT for the cluster by 1. In contrast, the DF for environmental surveillance depends on the number of individuals excreting poliovirus into the sewage system in a geographical subpopulation. We define the minimal number of effective infectiousness-weighted individuals who are infectious (EI) and compare this to the threshold (EI*) required for the system to detect the virus in the sewage. Moreover, we define s_i_ as the probability of detecting poliovirus in the i^th^ sewage sample given that EI>EI*. Each time when sample collection should occur (i.e., every month) we check whether the weighted number of excreting individuals exceeds the EI*.

We based our estimates for p values on review of the available AFP data focusing on confirmed and compatible cases and judgment of one of the expert authors (MAP). In northern India, while polio remained endemic, the detection of AFP cases occurred with oversampling, as indicated by a very high detection rate for AFP cases overall (i.e., 12.48/100,000 in India for 2013-14 compared to the GPEI standard of 2/100,000[5]). We account for the higher probability of relatively lower quality AFP surveillance in underserved areas by using relatively lower probabilities of detection in the subpopulations with lower immunization rates.

Given the conditions in northern India, we use constant high p_i_ values of 95% for detection of each case in the general population, and we use 80% for the under-vaccinated subpopulation. In contrast, even with endemic polio in northwest Nigeria, AFP surveillance has been less sensitive, with independent review teams finding cases of lameness most likely caused by polio that the system did not detect.[6] In northern Nigeria, we assume that the surveillance system for the general population misses relatively more cases than in India, but it improves following the detection of a cluster of cases such that the p values start low and increase within a cluster with p=(0.80, 0.85, 0.90, …, 0.90). For the under-vaccinated subpopulation in Nigeria, we assume one-third lower p values (i.e., 0.54, 0.57, 0.60, …, 0.60). Tajikistan represents an outbreak in a polio-free area, and for this outbreak we compared the number of compatible and confirmed polio cases as the outbreak occurred. We estimated the probabilities of detecting a case based on the number of compatible cases that preceded it. For example, if five compatible cases occurred before the first confirmed case, then we assume p_1_=1/6. Next, if three compatible cases occur between the first and second confirmed case, then p_2_=1/4. We continue this procedure until the first occurrence of at least two confirmed cases one after another, and we estimate the probability of detecting the later cases using the ratio of confirmed cases to the sum of confirmed and compatible cases listed from that point forward.

For environmental surveillance, we assume that after an initially relatively low probability of detection of a positive sample by environmental surveillance (i.e., s_1_=0.30), we assume that the system would increase the probability of detecting virus in all subsequent positive samples (i.e., s_2_=0.90) as long as circulation continues. Israel represents the only scenario we modeled that currently uses extensive environmental surveillance. We characterize EI* by modeled geographical region using population size, number of sampling sites per month from January 2013 to March 2014[7], and the detection level of 1 excreting individual per 10,000 inhabitants for each sampling site (see the bottom of Table 1).[8]

**A4. Population immunity figures**

Figure A1 shows the population immunity figures for the modeled populations.

**Figure A1:** Population immunity. Each dotted line (red, green, blue) represents the population immunity threshold EIP*=(1- 1/R0) for a given serotype, with red for type 1, green for type 2, and blue for type 3, respectively. Each solid line of the corresponding color presents the population immunity in the model for that corresponding serotype.

a) Northern India


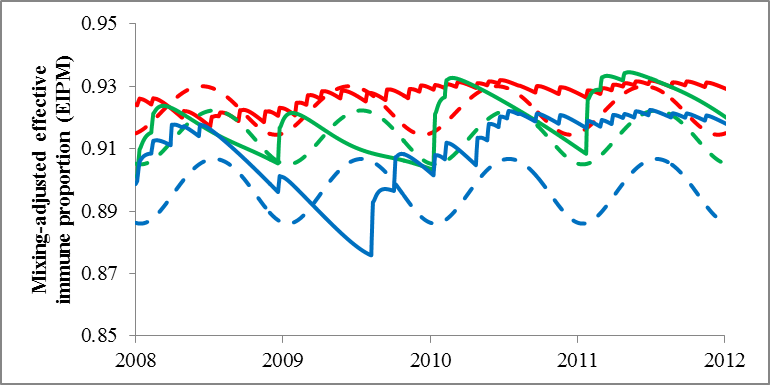


b) Northwest Nigeria 2014 current path


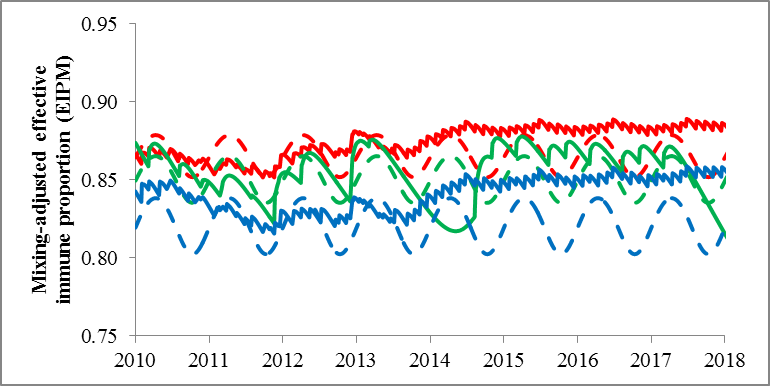


c) Northwest Nigeria increased tOPV


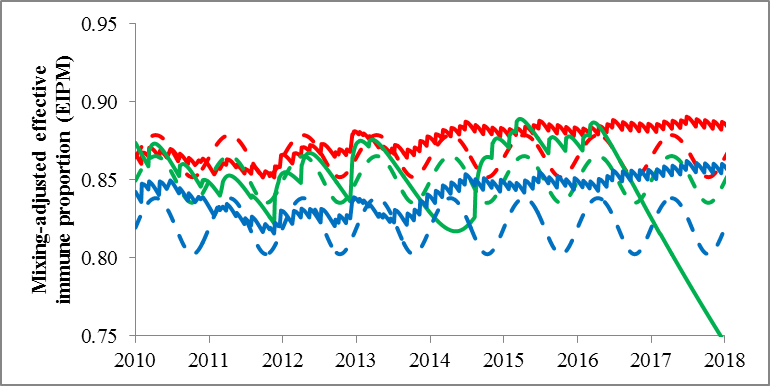


d) Tajikistan


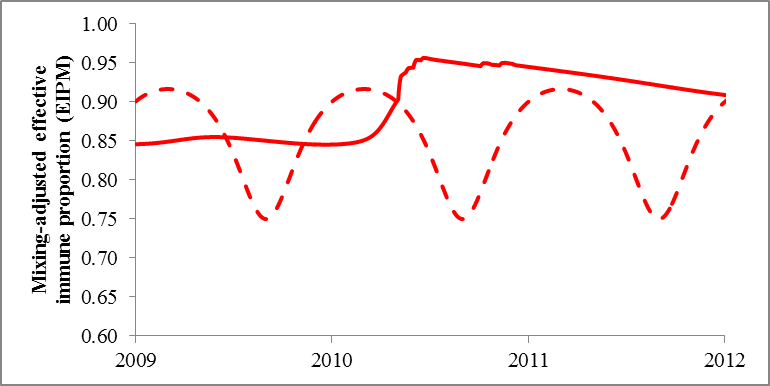


e) Israel


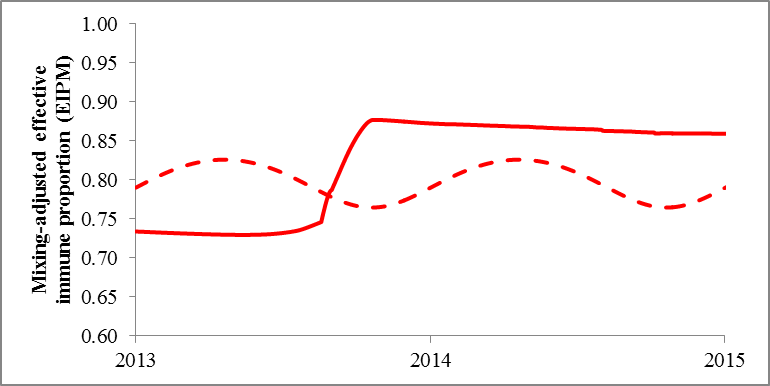


**References**

1. Kalkowska DA, Duintjer Tebbens RJ, Thompson KM: **Modeling strategies to increase population immunity and prevent poliovirus transmission in the high-risk area of northwest Nigeria**. *J Infect Dis* 2014, **210**(S1):412-423.

2. National Population Commission Nigeria and ICF International: **Nigeria Demographic and Health Survey 2013**. 2014.

3. Thompson KM, Duintjer Tebbens RJ: **Modeling the dynamics of oral poliovirus vaccine cessation**. *J Infect Dis* 2014, **210**(S1):475-484.

4. Duintjer Tebbens RJ, Thompson KM: **Modeling the potential role of inactivated poliovirus vaccine to manage the risks of oral poliovirus vaccine cessation**. *J Infect Dis* 2014, **210**(S1):485-497.

5. World Health Organization: **Conclusions and recommendations of the Advisory Committee on Poliomyelitis Eradication, Geneva, 27–28 November 2007**. *Wkly Epidemiol Rec* 2008, **3**(83):25-36.

6. Gidado SO, Nguku PM, Ohuabunwo CJ, Waziri NE, Etsano A, Mahmud MZ, Shuaib FM, Korir CK, Mkanda P, Bloland PB *et al*: **Polio Field Census and Vaccination of Underserved Populations — Northern Nigeria, 2012–2013**. *Morb Mort Wkly Rep* 2013, **62**(33):663-665.

7. Shulman LM, Mendelson E, Anis E, Bassal R, Gdalevich M, Hindiyeh M, Kaliner E, Kopel E, Manor Y, Moran-Gilad J *et al*: **Laboratory challenges in response to silent introduction and sustained transmission of wild type 1 poliovirus into Israel in 2013**. *J Infect Dis* 2014, **210**(S1):S304-314.

8. Hovi T: **The efficiency and reliability of polio surveillance**. *Dev Biol (Basel)* 2001, **105**:21-31.
